# Supplementary material for: Toward a study of gene regulatory constraints to morphological evolution of the Drosophila ocellar region
Source: Dev Genes Evol. 2016 Apr 1;226:221–33. doi: 10.1007/s00427-016-0541-8 (PMC4896973; doi:10.1007/s00427-016-0541-8)
Supplement: Supplementary file 1 — (PDF 221 kb) [file 427_2016_541_MOESM1_ESM.pdf]

Supplementary Data -Aguilar-Hidalgo et al.

Figure S1. Distribution of complementary distance (1-λ) between control CiA pattern and the one obtained when varying parameters in a two orders of magnitude range. Thresholds for a complementary distance 1-λ=0.8 ('good', blue), 1-λ=0.6 ('medium', red) and 1-λ=0.4 ('bad', yellow) are indicated.

Figure S1. Aguilar-Hidalgo et al.

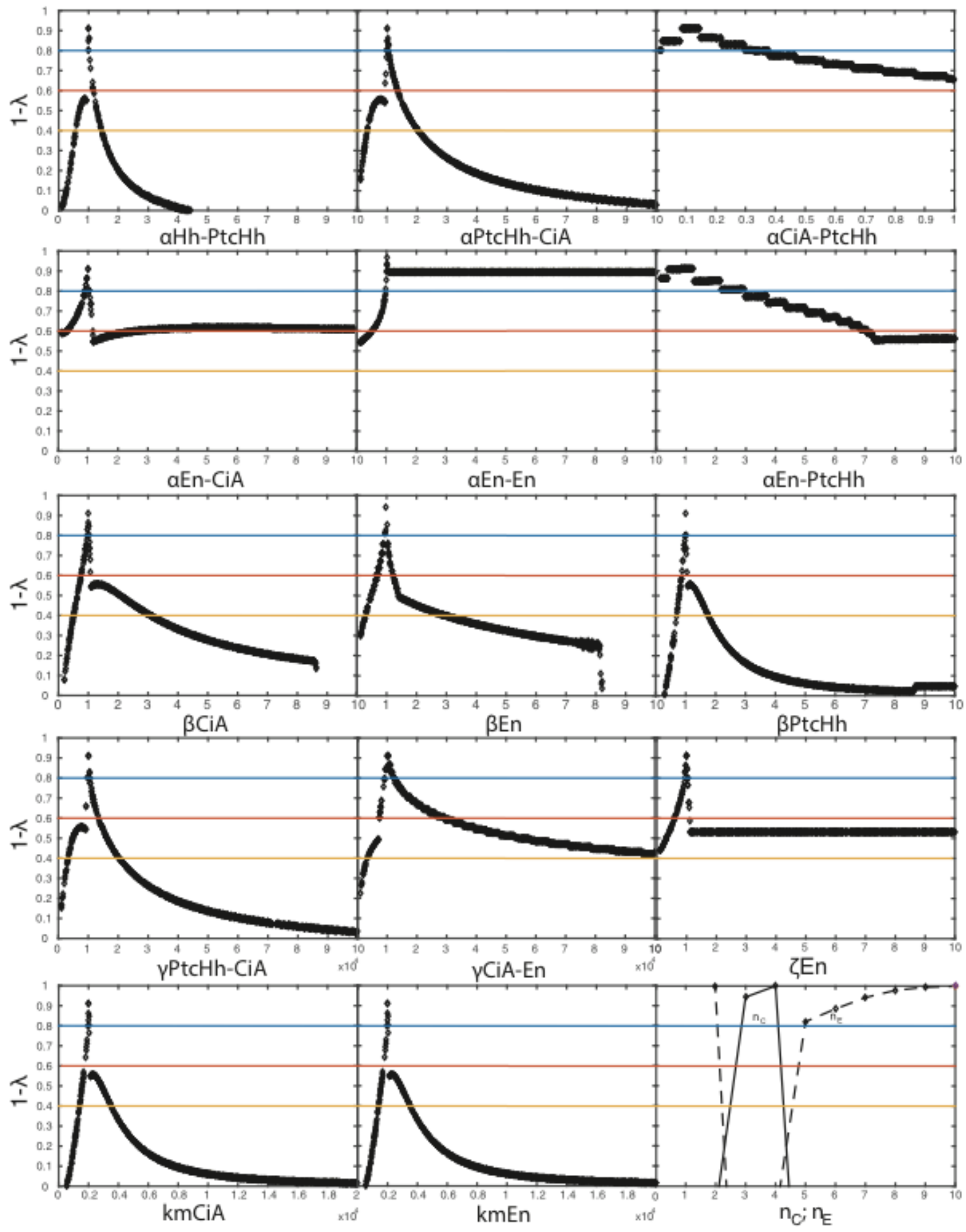

Figure S2. Simulations of the 3node-GRN (A) randomizing all parameters in the GRN network and (B) also including the Hh's effective diffusion *D* and effective Hh turnover β_{Hh}, which together define the shape of the Hh gradient. (S1) Reaction-diffusion equation describing the dynamics of Hh used in (B). The source term in this equation is described by a combination of Heaviside Theta functions Θ, which establish a source width ω centered at position x=0, with Hh production rate α_{hh}.

Figure S2. Aguilar-Hidalgo et al.

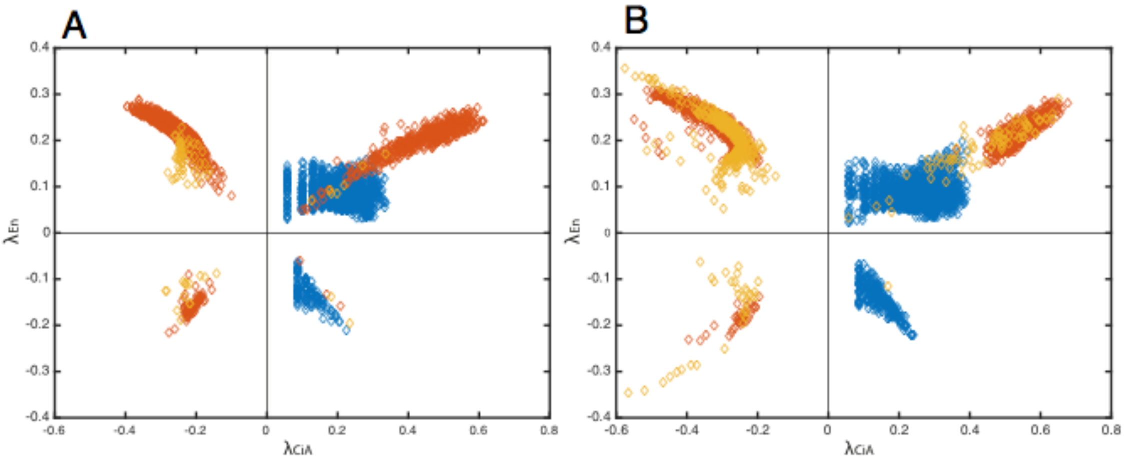

$$\frac{\partial\,[Hh]}{\partial t} = D\,\partial_x^2\,[Hh] - \beta_{Hh}\,[Hh] + \alpha_{hh}\,\Theta\left(\frac{\omega}{2} - x\right)\,\Theta\left(\frac{\omega}{2} + x\right) \qquad \textbf{(S1)}$$
